# Identification of a Synthetic Polyhydroxyphenolic Resveratrol Analogue, 3,3′,4,4′,5,5′-Hexahydroxy-*trans*-Stilbene with Anti-SARS-CoV-2 Activity

**DOI:** 10.3390/molecules28062612

**Published:** 2023-03-13

**Authors:** Walter Jäger, Eva Kicker, Melina Hardt, Riem Gawish, Pia Gattinger, Michaela Böhmdorfer, Sylvia Knapp, Rudolf Valenta, Kurt Zatloukal, Thomas Szekeres

**Affiliations:** 1 Department of Pharmaceutical Sciences, Division of Pharmaceutical Chemistry, University of Vienna, 1090 Vienna, Austria; 2 Diagnostic and Research Center for Molecular BioMedicine, Medical University of Graz, 8010 Graz, Austria; 3 Department of Medicine I, Research Division of Infection Biology, Medical University of Vienna, 1090 Vienna, Austria; 4 Department of Pathophysiology and Allergy Research, Center for Pathophysiology, Infectiology and Immunology, Medical University of Vienna, 1090 Vienna, Austria; 5 Clinical Institute for Laboratory Medicine, Medical University of Vienna, 1090 Vienna, Austria

**Keywords:** polyhydroxyphenol, SARS-CoV-2, COVID-19, antiviral effects

## Abstract

The Severe Acute Respiratory Syndrome Coronavirus 2 (SARS-CoV-2) virus has been causing the COVID-19 pandemic since December 2019, with over 600 million infected persons worldwide and over six million deaths. We investigated the anti-viral effects of polyphenolic green tea ingredients and the synthetic resveratrol analogue 3,3′,4,4′,5,5′-hexahydroxy-*trans*-stilbene (**HHS**), a compound with antioxidant, antitumor and anti-HIV properties. In the TCID_50_ assay, four out of nine green tea constituents showed minor to modest cell protective effects, whereas HHS demonstrated the highest reduction (1103-fold) of the TCID_50_, indicating pronounced inhibition of virus replication. **HHS** was also a highly effective inhibitor of SARS-CoV-2 proliferation in VeroE6 cells with an IC_50_ value of 31.1 µM. **HSS** also inhibited the binding of the receptor-binding domain (RBD) of the spike protein to the human angiotensin-converting enzyme 2 (ACE2) receptor (RBD-ACE2) binding with 29% at 100 µM and with 9.2% at 50 µM indicating that the SARS-CoV-2 inhibitory effect might at least in part be attributed to the inhibition of virus binding to ACE2. Based on the chemical similarity to other polyphenols, the oral bioavailability of **HHS** is likely also very low, resulting in blood levels far below the inhibitory concentration of **EGCG** against SARS-CoV-2 observed in vitro. However, administration of **HHS** topically as a nose or throat spray would increase concentrations several-fold above the minimal inhibitory concentration (MIC) in the mucosa and might reduce virus load when administered soon after infection. Due to these promising tissue culture results, further preclinical and clinical studies are warranted to develop **HHS** as an additional treatment option for SARS-CoV-2 infection to complement vaccines, which is and will be the main pillar to combat the COVID-19 pandemic.

## 1. Introduction

In December 2019, starting in Wuhan, China, a novel coronavirus (SARS-CoV-2) caused a pandemic that will continue until effective and safe vaccines can be provided to the world or causal drug treatment of the disease can help to avoid severe and fatal cases of the illness. Over 600 million persons have been infected worldwide, with increasing numbers who are awaiting additional effective treatment options. Although various vaccines and antiviral compounds were introduced, additional options to treat or prevent SARS-CoV-2 infections are warranted. 

Green tea polyphenols might be promising candidates as they exhibit antiviral activity against a wide range of diseases, including viral diseases. Epigallocatechin 3-gallate (**EGCG**) is the major ingredient in green tea and accounts for 50–80% of a brewed cup [1]. **EGCG** showed antiviral activity not only against influenza, hepatitis B and C, herpes simplex and HIV but also against SARS-CoV-2 [1]. It was also observed that **EGCG** had lowered the production of coronavirus-associated proteins in infected cells [1] and decreased the level of detected virus particles in a mouse model [2]. So far, no in vitro or in vivo data about the activity of other polyphenols in green tea against SARS-CoV-2 exist. However, molecular visualization and molecular docking experiments demonstrated the binding of **EGCG** and other green tea polyphenols to the central channel of the SARS-CoV-2 spike protein [3] and the SARS-CoV-2 main protease (Mpro) [4] as well as interactions with papain-like protease (PLpro), RNA-dependent RNA polymerase (RdRP), Helicase (nsp13) and the endonuclease Nsp15 (NendoU) [5].

**Figure 1:**
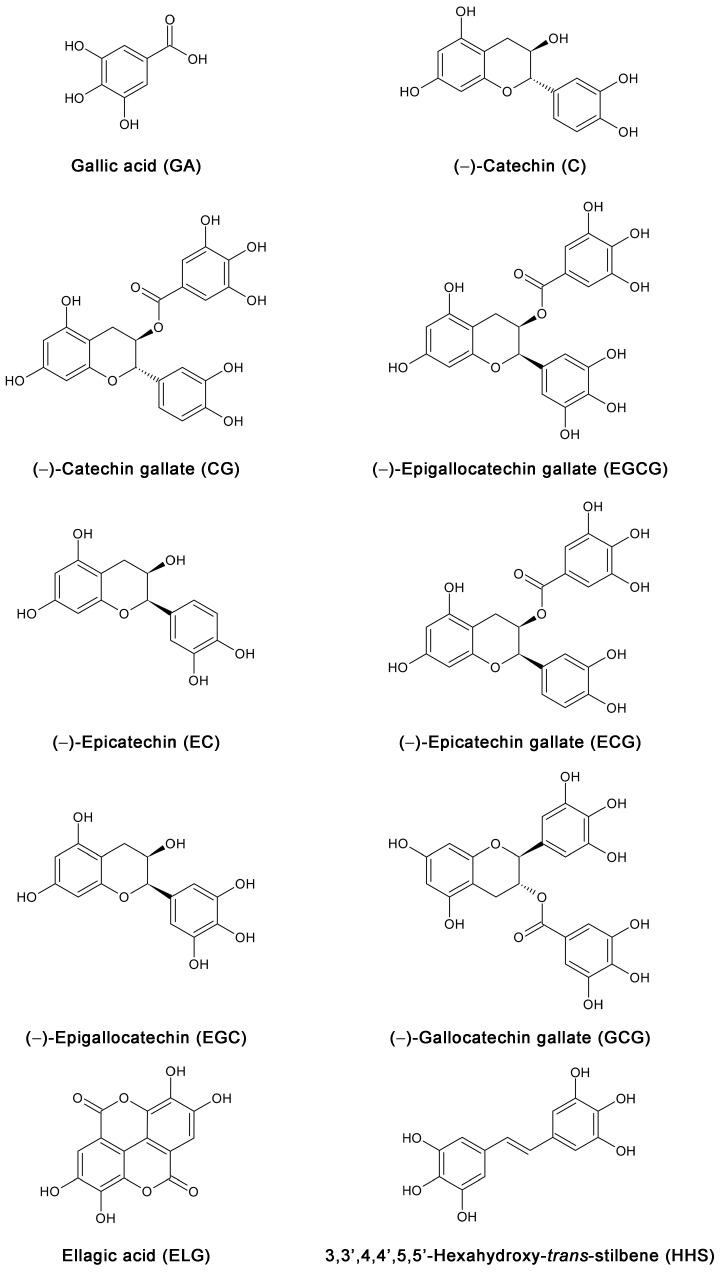
Chemical structures of the nine green tea constituents **GA**, **C**, **CG**, **EGCG**, **EC**, **ECG**, **EGC**, **GCG**, and **ELG**, as well as the synthetic hexahydroxy resveratrol analog **HHS** as a control compound, for structure–activity relationships.

We therefore investigated the effects of the main green tea catechins, namely catechin (**C**), catechin gallate (**CG**), **EGCG**, epicatechin (**EC**), epicatechin gallate (**ECG**), epigallocatechin gallate (**EGC**), and gallocatechin gallate (**GCG**) regarding their ability to inhibit or lower virus replication in vitro compared to the potent and clinically used anti-COVID-19 drug remdesivir. As Maiti and Banerjee [3] showed that the number of galloyl groups favors greater attachment to the receptor ACE2, we also included gallic acid (**GA**) and ellagic acid (**ELG**), a dimeric gallic acid derivative, in our study—both compounds also present in considerable amounts in green tea. Previous investigations in our lab demonstrated that the anticancer and anti-inflammatory effects of resveratrol analogues are increased by the number of OH groups [6,7,8]. In order to investigate whether the numbers and position of phenolic groups of green tea compounds may also correlate with activity, we included 3,3′,4,4′,5,5′-hexahydroxy-*trans*-stilbene (**HHS**), a synthetic resveratrol analogue. The chemical structures of all compounds used are shown in Figure 1.

As mentioned previously, **HHS** is an excellent free radical scavenger, thus inhibiting various enzymes crucial for nucleoside and nucleotide synthesis [9]. It inhibits ribonucleotide reductase, a key enzyme of nucleic acid synthesis [10]. In addition, **HHS** was shown to be a selective cyclooxygenase-2 inhibitor [7]. In vivo activity could be proved in a mouse melanoma model, showing anti-tumor activity and even inhibition of the development of melanoma metastasis [11]. Later **HHS** was shown by Han and coworkers to be an effective HIV-1 inhibitor [12]. They investigated this compound in different cell lines after infection with different strains of HIV-1. They found that **HHS** is acting at a very early stage of virus infection. It might inhibit a step prior to reverse transcription, possibly interacting with the entry of the virus into the target cells. The observed mechanism of action is different from the effects seen with resveratrol. Resveratrol competes with the substrates deoxynucleoside triphosphates (dNTPs), thus blocking chain polymerization [13]. 

As there are no in vitro data on whether **HHS** is able to inhibit virus uptake into cells, we further investigated the interaction of **HHS** with the angiotensin-converting enzyme 2 (ACE2) receptor, which is located on cell membranes and used by SARS-CoV-2 to gain entry into human cells. As host cell invasion is initiated through direct binding of the viral spike protein to ACE2, disrupting the spike-ACE2 interaction would be a potential therapeutic target for treating COVID-19. 

## 2. Results

### 2.1. Median Tissue Culture Infective Dose (TCID_50_) 

**Figure 2:**
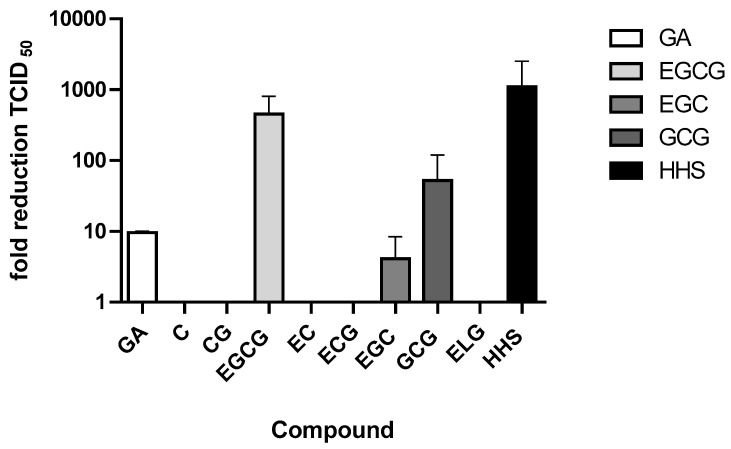
Effect of treatment of Vero cells with the respective substances compound (50 µM) after infection of the cells with SARS-CoV-2. Vero cells were preincubated for 45 min with either DMSO or the compound and infected with a SARS-CoV-2 stock. After five to seven days TCID50 dose was calculated. Values represent the average + half range of two determinations.

To investigate in a preliminary screen whether green tea constituents or HHS inhibit SARS-CoV-2 infection, VeroCCL81 cells were pretreated with the various compounds at a concentration of 50 µM, and the mixtures were subjected to TCID_50_ in order to determine virus titers. **GA**, **EGCG**, **EGC**, **GCG** and **HHS** showed cell protective effects and reduced the TCID_50_ by at least 1 Log scale. TCID_50_ was significantly lowered by 50 µM of **GA** (9.62 fold reduction), **EGCG** (451 fold reduction), **EGC** (4.09 fold reduction), **GCG** (52.4 fold reduction) and **HHS** (1103 fold reduction), indicating pronounced inhibition of virus replication. TCID_50_ -values for compounds **C**, **CG**, **EC**, **EGC** and **ELG** were below the detection limit and in the range of DMSO (Figure 2). In additional experiments, **GA**, **EGCG**, **EGC**, **GCG** and **HHS** were also incubated with non-infected Vero cells showing that the compounds did not interfere with cell growth, at least in concentrations up to 50 µM. 

### 2.2. Reduction of Virus Infection Efficiency

**Figure 3:**
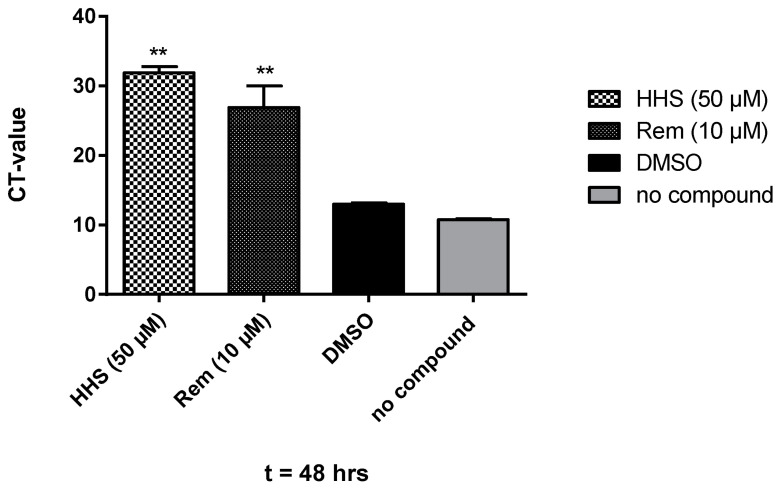
Treatment of Vero E6 cells with **HHS** (50 µM), remdesivir (10 µM) and DMSO (1% in medium). Compounds were present pre- and during infection with SARS-CoV-2 and during incubation. After 48 h, RNA was extracted, and the viral copy numbers were quantified via qRT-PCR. Values represent the CT values (means ± SD) of five determinations. Asterisks (**) indicate significantly different (*p* < 0.01) in comparison to no compound (non-parametric unpaired *t*-test).

As **HHS** showed the most pronounced inhibitor effects against SARS-CoV-2 in VeroCL81 cells, only this compound was used in further experiments but not the other minor potent green tea polyphenols. Figure 3 shows the effect of treatment with **HHS** on virus-infected VeroE6 cells. We observed a clear reduction of the amount of detected viral particles in the supernatant after 48 h in the presence of **HHS**; remdesivir, a clinically used anti-COVID-19 drug, served as a control. At 50 µM, **HHS** was highly effective, leading to a cycle threshold (CT) value of 31.9, indicating a 10^6^-fold reduction of infection and viral replication, a value significantly different from the untreated control (no compound). The CT value, therefore, shows the number of cycles necessary to spot the virus (the lower the CT value, the higher the viral load). Samples treated with remdesivir also showed a significant reduction of SARS-CoV-2 with a CT value of 26.9 at 10 µM. DMSO samples had no effect on the virus infection efficiency and were not significantly different from samples of the untreated control, indicating that DMSO (1%) does not interfere with the assay. This was further confirmed by a metabolic interference assay using non-infected VeroE6 cells resulting again in no significant changes in the metabolic activity of DMSO (1%) compared with untreated controls (the slopes k of linear regression analysis resulted in 89% metabolic activity; see Figure S1). We also investigated the possible cytotoxic properties of **HHS** against non-infected VeroE6 cells covering all conditions in which the virus neutralization assays were performed. As shown in Supplementary Materials Figure S2, **HHS** does not interfere with normal cell growth at 40 µM, 60 µM and even at 80 µM compared to DMSO (Figure S2). 

### 2.3. Inhibitory Concentrations of HHS

**Figure 4:**
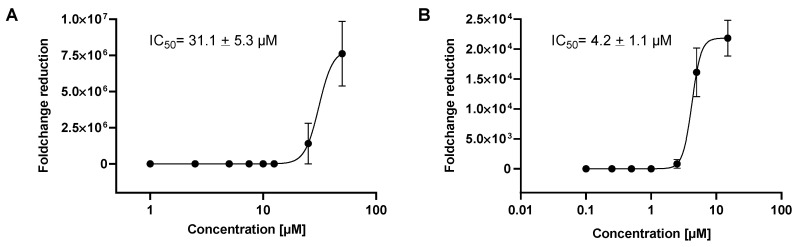
Inhibition of SARS-CoV-2 viral replication by **HHS** and remdesivir for 24 h, (**A**) HHS, (**B**) remdesivir. IC_50_ values are the concentrations where the viral infection was inhibited by 50%. Values were calculated by GraphPad Prism 9 and represent the means ± SD of three determinations.

**Figure 5:**
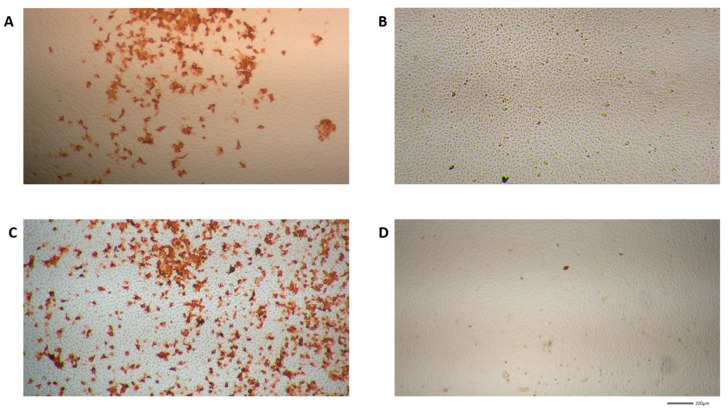
SARS-CoV-2 specific immunohistochemical staining (IHC) of VeroE6 cells treated with different concentrations of **HHS** and remdesivir as described in the methods part (images were made from IC_50_ experiments). Virus-infected cells appeared red. The microscopy images are shown in four-fold magnification. (**A**,**B**) **HHS** 7.5 µM and 50 µM; (**C**,**D**) remdesivir 0.25 µM and 10 µM.

In the next set of experiments, VeroE6 cells were seeded in 48 well plates, infected and incubated for 24 h with different concentrations of **HHS**, which showed the most pronounced reduction in virus replication in the previous experiments. **HHS** inhibited with an IC_50_ of 31.1 µM, and remdesivir inhibited 50% of virus proliferation at 4.2 µM (Figure 4). Inhibition of virus proliferation could also be confirmed by immunohistochemical (IHC) staining with an anti-SARS-CoV-2 nucleocapsid-specific antibody, as depicted in Figure 5. Infected cells appeared red, and a clear dose dependency of viral inactivation was visible. Treatment of the cells with 7.5 µM **HHS** and 0.25 µM remdesivir still led to the infection of numerous cells, whereas 50 µM **HHS** and 10 µM remdesivir showed a clear viral inhibiting effect; only single cells were stained positive in a confluent cellular monolayer. 

### 2.4. Inhibition of Virus Uptake

**Figure 6:**
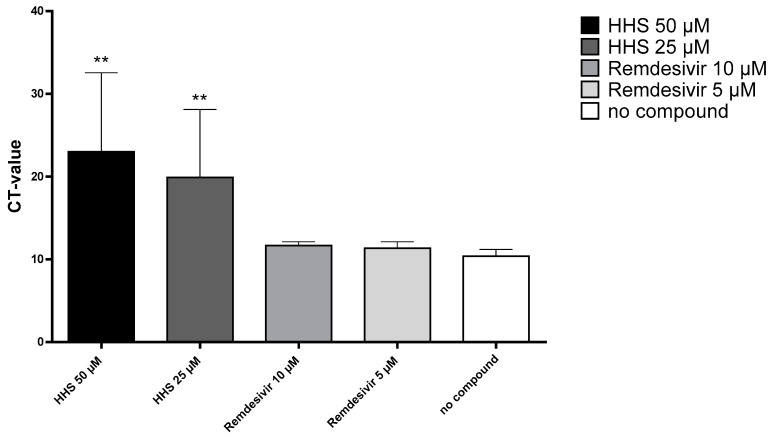
Incubation of VeroE6 cells with **HHS** and remdesivir for one hour in parallel to virus infection. Compounds were then washed away and the cells were further incubated in medium (MEM 2% FCS) only. After 24 h, the cell supernatant was removed and tested by qPCR as described above. Values for the viral uptake inhibition represent the CT values (means ± SD) of five determinations. Asterisks (**) indicate significantly different (*p* < 0.01) in comparison to no compound (Kruskal–Wallis multiple comparisons).

In order to determine whether inhibition of virus replication was due to alterations of virus uptake into the host cells and/or inactivation of the virus, we incubated the cells with **HHS** for one hour in the presence of the virus. **HHS** could inhibit virus replication in a concentration-dependent manner after short-term incubation for one hour, as evidenced by significantly increased CT after incubation with 25 or 50 µM compared to no compound control (results are shown in Figure 6). These results indicate that virus uptake into the host cell or viral integrity could be inhibited by **HHS**. Remdesivir, which acts as a nucleoside analogue, showed no inhibitory effect during the one-hour infection period (CT values: 11.7 and 11.4 for 10 µM and 5 µM). 

### 2.5. Inhibition of ACE2 Binding

**Figure 7:**
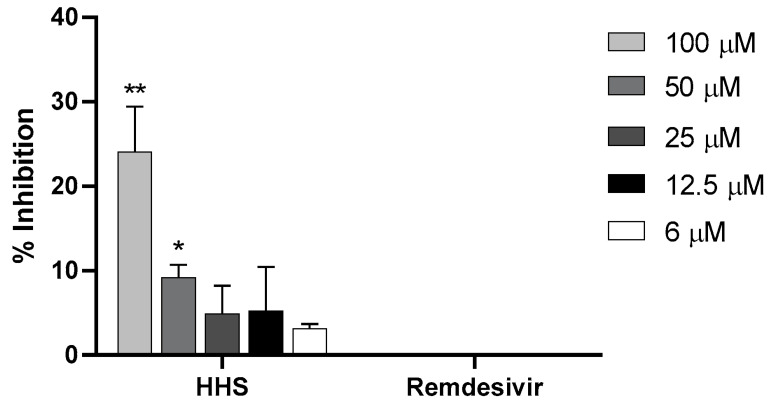
Percentages of inhibition of RBD-ACE2 binding (y-axes) after pre-incubation for 3 h at RT with various concentrations of **HHS** and remdesivir (x-axes) followed by a 3 h overlay onto plate-bound ACE2. Bound RBD was detected with a mouse monoclonal anti-His antibody followed by a HRP-labelled anti-mouse IgG_1_ antibody and detected with ABTS. Values represent the means ± SD of three determinations. Asterisks (*, **) indicate significantly different (*p* < 0.5 and *p* < 0.01, respectively) in comparison to 6 µM **HHS** (non-parametric unpaired *t*-test).

We then tested if remdesivir or **HHS** were able to inhibit RBD-ACE2 binding. As shown in Figure 7, remdesivir had no inhibitory effect on the binding to the receptor. However, **HHS** could inhibit binding to ACE 2 in a concentration-dependent manner (Figure 7). At 100 µM, **HHS** significantly inhibited ACE2 binding with 29% and at 50 µM with 9.2% compared to the lowest **HHS** concentration (6 µM). Even at low concentrations of 25 µM, 12.5 µM and 6 µM, inhibitions rates of 4.9, 5.2 and 3.1 percent were seen. However, based on the high standard deviations at 12.5 µM and 25 µM, these concentrations did not show significance to 6 µM. We, therefore, conclude that **HHS** can also inhibit virus uptake into the cell at a very early stage, which, at least in part, could be attributed to the blocking of the RBD-ACE2 binding.

## 3. Discussion

In the present study, we screened nine green tea polyphenols against SARS-CoV-2. Up to now, only the main constituent **EGCG** was shown to be active against infected Vero cells. For minor green tea constituents, only modeling and data, e.g., against the central channel of the SARS-CoV-2 spike protein, the proteases Mpro, Plpro, RdRP, and nsp13 and Nsp15 are available so far [3,4,5].

Calculating the median tissue culture infective dose of TCID_50_, four out of nine green tea compounds were active (Figure 2). The structure–activity relationships revealed that **EGCG**, **EGC** and **GCG** contain a pyrogallol group in their chemical structure (pyrogallol: benzene 1,2,3, triol is a decarboxylation product of gallic acid). Compounds with only one galloyl group (CG and **ECG**), however, showed no virus inhibition. Interestingly, **GA** was slightly active, possibly because of the additional carboxyl group. One galloyl and one pyrogallol group further increased the potency (13-110-fold reduction), as shown for GCG and **ECGC**. **HHS**, a synthetic resveratrol analog containing two pyrogallol groups, showed the highest fold reduction of viral replication (1103-fold), indicating that pyrogallol groups are more important for activity than galloyl groups. Our data are in contrast to Maiti and coworker [3], who did not discriminate between the galloyl and pyrogallol groups and, therefore, wrongly stated that the numbers of galloyl groups of catechins favor binding in the central channel of the spike protein. However, we are well aware that in addition to galloyl and pyrogallol groups, other structural features of green tea constituents, e.g., lipophilicity, molecular weight, and binding affinities to SARS-CoV-2 proteins, may also contribute to the activity. 

Next, we elucidated the effect of **HHS** (the compound which proved most effective in a previous experiment) on the amount of detected viral particles of infected VeroE6 cells. Again, **HHS** at 50 µM was very effective, with a CT value above 30 (Figure 3). As expected, remdesivir was more potent than **HHS**, showing a CT value of 26.9 already at 10 µM leading to an IC_50_ value of 4.2 µM compared to that of 31.1 µM for HHS. The IC_50_ values of **HHS** in the Vero cells are much higher than in HL-60 leukemia cells, as **HHS** is inhibits key enzymes of DNA synthesis, thus specifically targeting rapidly proliferating malignant cells [9]. The IC_50_ for remdesivir in our experiments was 4.2 and only slightly higher than what was determined by Pruijssers et al. with a value of 1.65 µM [14]. This difference might be due to different assay parameters and the use of infected lung cells instead of Vero cells. 

**HHS** incubation for only one hour could also effectively inhibit virus replication, as shown in Figure 6. This suggests that **HHS** might block virus entry into the cells, at least to a certain extent. An interaction between HHS and the viral envelope may also be a possible mode of action, as inhibitory effects in this setup likely occur before or during viral attachment to the cell surface or in a very early uptake state. As **HHS** acts through different mechanisms, mutations of the virus could be sensitive toward treatment with **HHS**. We hypothesize that due to its unique mechanisms of action, **HHS** can also be used successfully in combination with other antiviral compounds to target certain steps of virus proliferation for early treatment of SARS-CoV-2 infections. 

In a final experiment, we tested whether **HHS** might inhibit RBD-ACE2 binding. Indeed, **HSS** showed a partly inhibitory effect on the binding to the receptor (29% inhibition at 50 µM and 9.2% at 25 µM). In addition to binding to the ACE2 receptor, further mechanisms inhibiting nucleic acid synthesis or inhibition of proteases [1,15], such as 3C-like protease, the main protease found in coronaviruses, might also contribute to the observed inhibition of virus entry into VeroE6 cells. Remdesivir showed no binding to ACE2 as it exerts its antiviral activity inside the host cell, where it is metabolized into nucleotide triphosphate (NTP). NTP then suppresses viral replication by targeting the RNA-dependent RNA polymerase [16]. 

Until now, there are no data about peroral absorption, distribution, and metabolism of **HHS** in animal models or humans. However, based on two pyrogallol groups, oral bioavailability should be low and comparable to the green tea polyphenol **EGCG**. Even at a high dose of **EGCG** (375 to 1200 mg), maximum blood levels were only 4.3–5.6 μM and far below the inhibitory concentration of **EGCG** against SARS-CoV-2 observed in vitro [17]. However, since SARS-CoV-2 particles accumulate in epithelial cells of the saliva glands and oral mucosa, **HHS** could be applied as a throat spray, which should lead to concentrations several-fold above the minimal inhibitory concentration (MIC). Indeed, in a very recent study, human volunteers applied two puffs of green tea throat spray (8% green tea extract), and throat swaps were taken from the pharyngeal region of each participant before and after spray application [18]. The median **ECGC** amount in the saliva 30 min after spray application was very high and ranged between 344 and 407 µg/mL saliva (740 µM and 888 µM). 

In conclusion, our data demonstrated that the synthetic polyphenolic compound HSS is an effective inhibitor of SARS-CoV-2 infection in vitro. **HHS** also inhibited ACE2-RBD binding, indicating that its SARS-CoV-2 inhibitory effect could be at least in part attributed to the ACE2-RBD binding. As polyphenols are extensively metabolized in the gut and liver after peroral administration, **HHS** might therefore be locally used as a throat spray or be inhaled to prevent bronchial virus proliferation and thus prevent severe illness after SARS-CoV-2 infection. Animal models and patient data would have to confirm our in vitro results. Further preclinical and clinical studies are therefore warranted to develop **HSS** as a promising treatment option for SARS-CoV-2 infection. 

## 4. Materials and Methods

### 4.1. Chemicals 

**GA**, **C**, **CG**, **EGCG**, **EC**, **ECG**, **EGC**, **GCG** and **ELG** with purities of ≥95% were obtained from Sigma-Aldrich, Munich, Germany. DMSO for molecular biology (99.9%) was purchased from Merck KgaA (Darmstadt, Germany). Remdesivir (MCE HY-104007) was obtained from THP Medical Product, Vienna, Austria. **HSS** was provided by the Department of Pharmaceutical Sciences, University of Vienna, Austria.

### 4.2. Median Tissue Culture Infective Dose (TCID_50_) Assay

The human SARS-CoV-2 isolate BetaCoV/Munich/BavPat1/2020 (referred to as BavPat1) was kindly provided by Christian Drosten, Charité, Berlin, and proliferated in VeroCCL81 cells. Infected VeroCCL81 cells were then plated in growth medium (DMEM containing 10% fetal calf serum (FCS), 100 µM non-essential amino acids, 1 mM sodium pyruvate and penicillin/streptomycin) into a 96-well plate at a density of 1.5–2 × 10^4^/well on the evening before the experiment. On the next day in the morning, the growth medium was removed, and 95% confluent Vero cells were preincubated for 45 min with either DMSO or **GA**, **C**, **CG**, **EGCG**, **EC**, **ECG, EGC**, **GCG**, **ELG** and **HHS** at a dose of 50 µM and infected with 100 µL of serial dilutions of a SARS-CoV-2 virus stock (serial ten-fold dilutions; range from 1^10^–10^8^). Preincubation and virus infection was carried out in a medium containing only 2% FCS instead of 10% FCS, as previous data showed interference with the assay by the containing scavenging substances. In contrast to a more precise plaque assay, the number of plaque-forming units was not counted, but wells were only classified as infected (dead cells) or uninfected (healthy cells) for each dilution step. After five to seven days (no differences in the results between these two days were seen) all wells were checked for cell viability by light microscopy, and the TCID_50_ dose in the presence or absence of indicated compounds was calculated according to the Reed and Munch method [19]. Wells with viable and infected cells were identified using an inverse light microscope showing either an intact monolayer of Vero cells (viable) or wells with a massive CPE (cytopathic effect), characterized by a large number of rounded or detached cells and the absence of a monolayer (infected). 

### 4.3. Virus Neutralization Assay

The human 2019-nCoV isolate (Ref-SKU: 026V-03883 (Wuhan Strain) obtained from Charité Berlin was proliferated in VeroE6 cells (African green monkey kidney cells purchased from Biomedica, Vienna, Austria; VC-FTV6), TCID50 titers were determined according to the Reed Munch method [19]. Plaque-forming units (PFU) were calculated with a conversion factor of 0.7, based on the ATCC-LGC Standards (www.atcc.org/support/technical-support/faqs/converting-tcid-50-to-plaque-forming-units-pfu). For virus neutralization experiments, the working stocks were diluted to a calculated multiplicity of infection (MOI) 0.002 in minimal essential medium (MEM) supplemented with 2% FCS. Experiments with active SARS-CoV-2 virus samples were performed under BSL-3 conditions. VeroE6 cells were seeded at a density of 3.0E + 04 cells per well in a 48-well plate (Corning Costar (Corning, NY, USA), cell culture treated) in 300 µL (MEM) + 2% FCS, 24 h prior to the virus neutralization assay. On the infection day, the seeding medium was removed, a medium change was made, and the pre-diluted test compounds (60 mM to 20 mM) were diluted 1:100 directly in the well containing medium and mixed properly. The final compound concentration in the well ranged from 60 µM to 20 µM. This was made 30 min prior to the viral infection. During the one-hour infection with the virus diluted to a calculated MOI of 0.002, cells were kept at 37 °C and 5% CO_2_. After that, the infection medium was removed, and the cells were washed twice with MEM w/o supplements to remove the unbound virus. Subsequently, fresh MEM + 2% FCS was added, and **HHS** was diluted 1:100 in the well and mixed properly. The cells were incubated for an additional 48 h at 5% CO_2_ and 37 °C until 140 µL supernatant was harvested and inactivated with AVL (viral lysis) buffer to extract RNA and quantify the viral copy numbers via quantitative real-time polymerase chain reaction (qRT-PCR). Untreated infected cells served as positive controls in the assay; no additional compounds to lower or inhibit the viral replication were used in these controls. Non-infected cells served as the background reference of the assay; no compounds and no viruses were used in the negative controls. Remdesivir was used as a control with known antiviral activity [14]. In addition, the 48-well plate was fixed in 4% formaldehyde for SARS-CoV-2-specific immunohistochemical staining (IHC). 

### 4.4. Metabolic Activity (MA) Assay

VeroE6 Cells were seeded in 48-well plates with a 3.0E + 04 cells per well in 300 µL MEM supplemented with 2% FCS at 37 °C and 5% CO_2_. After 24 h, the medium was changed, and cells in MEM + 2% FCS were exposed to 1% DMSO or HHS (40, 60, and 80 µM final concentration in 1% DMSO) and further incubated for 1, 24, or 48 h. Then cells were washed twice with MEM without supplements, and the metabolic activity was measured over 120 min using a resazurin-based assay (BioTek Plate Reader Synergy 4, Thermo Fisher Scientific, Waltham, MA, USA). After the addition of resazurin (10 µM), the increase of relative fluorescent unit (RFU) was measured at 485/590 nm and a linear regression analysis was performed. The slopes (k) from 1% DMSO-treated cells were compared and normalized to the untreated controls or to **HHS**-treated cells (40, 60 and 80 µM), respectively. From the slopes of the linear regression, the relative metabolic activity (RMA) was calculated.

### 4.5. RNA Isolation, qRT-PCR and Determination of Viral Copy Numbers 

Viral RNA was isolated using the QIAamp viral RNA Mini Kit (QIAGEN, Hilden, Germany) following the manufacturer’s protocol. The viral RNA was then amplified with the CDC-recommended primers and probe set of N1 and N2 from 2019-Novel Coronavirus (2019-nCoV). A real-time qRT-PCR Panel was conducted (Table 1) using a QuantiTect Multiplex RT-PCR Kit (QIAGEN, Hilden, Germany) with a Rotor Gene Q cycler. The reaction volume was reduced to 25 µL with amplification for 30 min. at 50 °C and 15 min. at 95 °C; the steps were followed by 45 cycles (95 °C for 3 min. and 55 °C for 30 s). Table 1 lists the sequences of the used primers and probes. The calculation of viral copy numbers was performed identically to the protocol described by Kicker et al. [18]. Briefly, a commercially available copy number standard (ATCC VR-1986D genomic RNA from 2019 Novel Coronavirus, Lot: 70035624) was serially diluted and analyzed via qRT-PCR. The resulting CT values were plotted against ln[copy numbers], and the equation received from linear regression analysis was used to calculate the viral copy numbers from the CT values of the samples for Primer and Probe N1 and N2, respectively. Regression equations obtained for N1: y = −1.442 x + 35.079 and N2: y = −1.5 x + 38.357. These calculated copy numbers and measured CT values refer to a volume of 5 µL RNA eluate, which was used as a template in qRT-PCR. 

### 4.6. Immunohistochemistry (IHC) 

Immunohistochemistry was performed as described previously [18]. Briefly, after fixation of the cells with 4% formaldehyde, cells were permeabilized using 0,1% Triton X 100 in PBS for 10 min, and the endogen peroxidases were blocked with 3% H_2_O_2_ in methanol for 30 min. After three washing steps with PBS, the cells were incubated for 1 h with a 1:1000 dilution of primary antibody (SARS-CoV-2 (2019-nCoV) Nucleocapsid Antibody (Rabbit Mab, Sinobiological Cat: 40143-R019) in antibody diluent (REAL Antibody diluent, Agilent Technologies, Santa Clara, CA, USA, Dako Cat: S202230_2) followed by the treatment with the secondary antibody (EnVision™ + Dual Link System HRP, Agilent Technologies, Dako Cat: K5007). After washing (PBS 3x), the substrate (AEC substrate-Chromogen, Agilent Technologies, Dako, Cat: K346430-2, 2 drops) was dropped on the cells and incubated until viral infected cells were stained red. The reaction was then stopped with washing in PBS (3x) and wells were kept humid until photo documentation. For photo documentation, fourfold magnification with a Nikon Eclipse TS100 microscope (Nikon, Japan, Tokyo) was used with the JENOPTIK GRYPHAX^®^ camera and software (Jena, Germany) were used.

### 4.7. Inhibition of ACE2 Binding

In addition to classical virus neutralization tests, molecular interaction assay (MIAs) allow to test components for their specific ability to inhibit the binding of RBD from SARS-CoV-2 to its cognate receptor ACE2 [20]. The MIA to detect the inhibition of RBD to ACE2 receptor binding was performed as described [21,22] with the following alterations. An amount of 200 ng His-tagged RBD was incubated with various doses (100, 50, 25, 12.2, and 6 mM) of **HHS**, **EGCG**, **GCG**, and remdesivir for three hours at RT followed by a 3 h overlay onto plate-bound human ACE2 receptor protein (2 µg/mL). Bound RBD was then detected with a mouse monoclonal anti-His antibody followed by a HRP-labelled anti-mouse IgG_1_ antibody and detected with (ABTS). All measurements were performed in duplicate with a variation of <5%.

### 4.8. Statistical Analysis

Statistical analyses were performed using GraphPad Prism software for Windows, version 9.0 (San Diego, CA, USA). For the calculation of *p*-values, either one-way ANOVA (non-parametric) with multiple comparison test (Kruskal–Wallis test) when *n* = 5 or unpaired test (non-parametric: Mann–Whitney test) when *n* = 3. Differences with *p* < 0.05 are considered statistically significant. Unless stated otherwise, all data are shown as means ± standard deviation (SD). The IC_50_ values were calculated as nonlinear regression: Log (inhibitor) vs. response (two parameters) constrain equal to 0.

## Figures and Tables

**Table 1:** qRT-PCR primers and probes.

Name	Description	Sequence (5′ > 3′)	Label
2019-nCoV_N1-F	2019-nCoV_N1 Forward Primer	GAC CCC AAA ATC AGC GAA AT	none
2019-nCoV_N1-R	2019-nCoV_N1 Reverse Primer	TCT GGT TAC TGC CAG TTG AAT CTG	none
2019-nCoV_N1-P	2019-nCoV_N1 Probe	FAM-ACC CCG CAT TAC GTT TGG TGG ACC-BHQ1	FAM, BHQ-1
2019-nCoV_N2-F	2019-nCoV_N2 Forward Primer	TTA CAA ACA TTG GCC GCA AA	none
2019-nCoV_N2-R	2019-nCoV_N2 Reverse Primer	GCG CGA CAT TCC GAA GAA	none
2019-nCoV_N2-P	2019-nCoV_N2 Probe	FAM-ACA ATT TGC CCC CAG CGC TTC AG-BHQ1	FAM, BHQ-1

## Data Availability

Not applicable.

## Supplementary Materials

The following supporting information can be downloaded at: https://www.mdpi.com/article/10.3390/molecules28062612/s1; Figure S1: Metabolic activity of VeroE6 cells incubated with DMSO (1%) for 48 h; Figure S2: VeroE6 cells were incubated with HHS (40–80 µM) for 1 h (washed and incubated in MEM + 2 % FCS for additional 24 h), 24 h or 48 h.
