# Supplementary material for: Identification of a Synthetic Polyhydroxyphenolic Resveratrol Analogue, 3,3′,4,4′,5,5′-Hexahydroxy-trans-Stilbene with Anti-SARS-CoV-2 Activity
Source: Molecules. 2023 Mar 13;28(6):2612. doi: 10.3390/molecules28062612 (PMC10056577; doi:10.3390/molecules28062612)
Supplement: Supplementary file 1 [file molecules-28-02612-s001.zip › molecules-2245283-supplementary.pdf]

Supplementary Materials

Identification of a synthetic polyhydroxyphenolic resveratrol analogue, 3,3',4,4',5,5'-hexahydroxy-*trans*-stilbene with anti SARS-CoV-2 activity

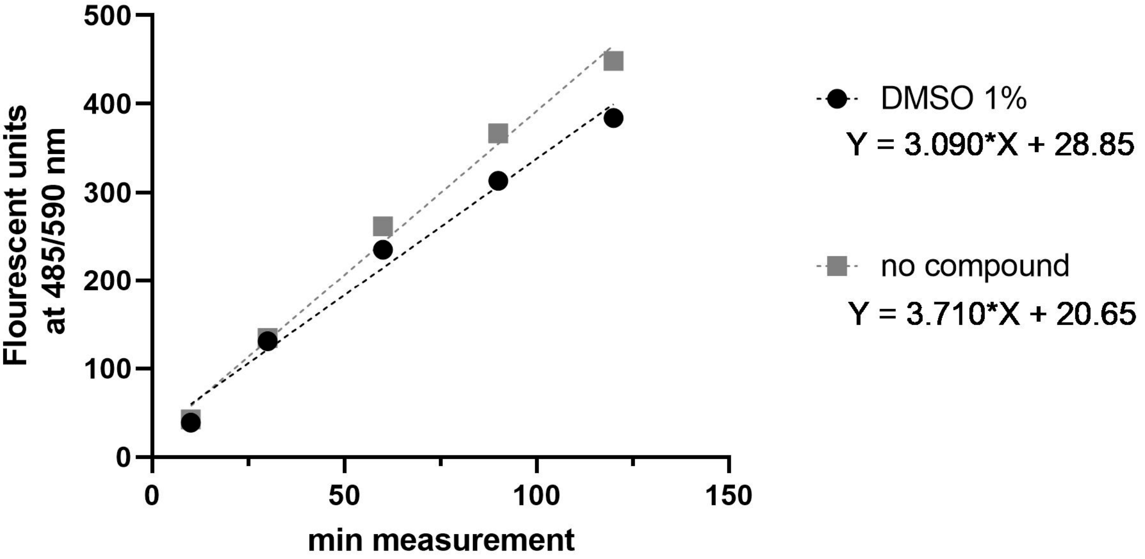

Figure S1. Metabolic activity of VeroE6 cells incubated with DMSO (1%) for 48 h. After washing the cells metabolic activity was measured over 120 min at wavelength of 485/520 nm. Data are presented as mean value of two determinations.

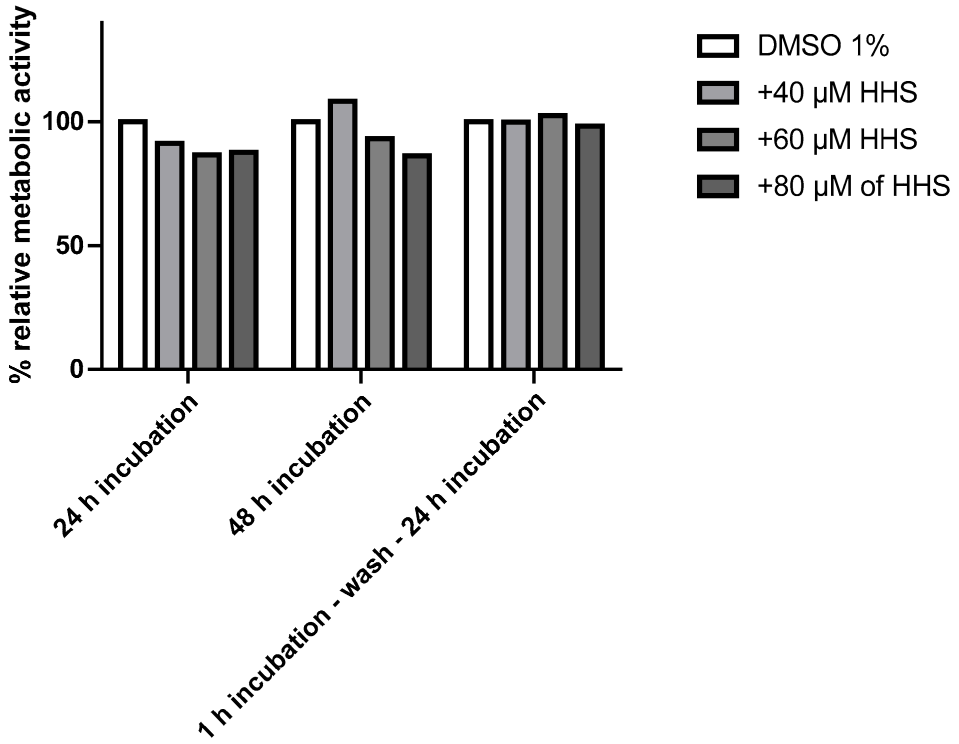

Figure S2. VeroE6 cells were incubated with HHS (40-80 µM) for 1 h (washed and incubated in MEM + 2 % FCS for additional 24 h), 24 h or 48 h. Metabolic activity was measured over 120 min at wavelength of 485/520 nm. Data are presented as mean value of two determinations
